# Hb Levels and Sex Differences in Relation to Short-Term Outcomes in Patients With Acute Myocardial Infarction

**DOI:** 10.3389/fcvm.2021.653351

**Published:** 2021-07-16

**Authors:** Junyu Pei, Xiaopu Wang, Pengfei Chen, Keyang Zheng, Xinqun Hu

**Affiliations:** ^1^Department of Cardiovascular Medicine, The Second Xiangya Hospital, Central South University, Changsha, China; ^2^Department of Cardiovascular Medicine, Beijing Anzhen Hospital, Capital Medical University, Beijing, China

**Keywords:** sex difference, hemoglobin levels, acute myocardial infarction, major bleeding, stroke, cardiovascular death, major adverse cardiovascular events

## Abstract

**Background:** Women had worse outcomes after acute myocardial infarction (AMI), and physiologically, women had lower hemoglobin values. We examined whether there were sex-related differences in the relationship between hemoglobin levels and adverse outcomes in patients with acute myocardial infarction.

**Method:** We conducted a *post-hoc* analysis of data from the Acute Coronary Syndrome Quality Improvement in Kerala (ACS-QUIK) Study. We explored the relationship between baseline hemoglobin level and 30-days adverse outcomes by logistic regression model, generalized additive model (GAM) and two-piecewise linear regression model. We used multiple imputation, based on five replications and a chained equation approach method in the R multiple imputation procedure, to account for missing data. The primary outcome were 30-day major adverse cardiovascular events (MACEs) defined as death, reinfarction, stroke, and major bleeding. The secondary outcomes were 30-day major bleeding, 30-day stroke and 30-day cardiovascular death (CVD death).

**Results:** Twenty thousand, five hundred fifty-nine patients with AMI were included in our analysis. Baseline hemoglobin level was associated with major bleeding [OR: 0.74, 95%CI (0.60, 0.92) *P* < 0.01], CVD death [OR: 0.94, 95%CI (0.90, 0.99) *P* < 0.01], and MACEs [OR: 0.95, 95%CI (0.92, 0.99) *P* < 0.01]. There was no significant relationship between baseline hemoglobin level and stroke incidence in both men [OR: 1.02, 95%CI (0.90, 1.14) *P* = 0.77] and women [OR: 1.15, 95%CI (0.96, 1.37) *P* = 0.18]. Baseline hemoglobin level was associated with major bleeding [OR: 0.71, 95%CI (0.58, 0.85) *P* < 0.01] in male patients, however we did not find the same relationship in female patients [OR: 0.89, 95%CI (0.56, 1.41) *P* = 0.61]. GAM and two-piecewise linear regression model showed the relationships of hemoglobin level with major bleeding, CVD death, and MACEs were non-linear (non-linear *P* < 0.05), and the threshold value were 13, 14.8, and 14.3 g/dL for MACEs and CVD death, respectively.

**Conclusion:** Baseline hemoglobin level was one of the independent predictors of prognosis in South Asia patients with acute myocardial infarction. Moreover, its impact on prognosis was largely different depending on the patients' sex.

## Background

Anemia is a very common comorbidity in patients with acute coronary syndrome (ACS). In previous studies, the prevalence of anemia in patients with acute coronary syndrome was 10–32% (1–4). Many cohort studies and *post-hoc* analysis of randomized controlled trials reported anemia was associated with hemorrhagic events and ischemic events in ACS patients (1, 5–11). Leonardi et al. reported that among patients with ACS managed invasively, in-hospital hemoglobin drop ≥3 g/dl, even in the absence of overt bleeding, is common and is independently associated with increased risk for 1-year mortality (12). In a recent meta-analysis involving 233,144 patients pointed out that anemia in patients with ACS was independently associated with a significantly increased risk of early and late mortality (1, 13). According to the Injuries and Risk Factors (GBD) 2010 Study, global anemia prevalence in 2010 was 32.9%, prevalence in females was higher, and south Asia had the highest burden (14). Women accounted for 30% of ACS patients, and were always older and sicker than men. Recent studies also found sex differences in baseline characteristics, prognosis, and management of ACS (15).

In high-income countries, the decline of mortality rates was observed in patients with ACS since 1970s (16). However, 80% of the world's coronary heart disease (CHD) deaths occurred in low and middle-income countries (LMIC), especially in South Asia (17). In India, CHD accounted for about 40% of urban deaths and 30% of rural deaths (18).

Recent studies did not have data on South Asian populations, and most of the data sources came from 10 years ago (19, 20). Considering the technological and diagnostic innovations of the past decade, our study was expected to provide more evidence of the impact of hemoglobin on prognosis in ACS population of Asian developing countries by using data from Acute Coronary Syndrome Quality Improvement in Kerala (ACS-QUIK) Study. Furthermore, the influence of sex difference on prognosis in the relationship between hemoglobin level and AMI was also discussed in this population.

## Method

### Study Participants and Data Collection

We conducted a *post-hoc* analysis of data from the Acute Coronary Syndrome Quality Improvement in Kerala (ACS-QUIK) Study (data obtained from the Biologic Specimen and Data Repository Information Coordinating Center, National Heart, Lung and Blood Institute, U.S. Department of Health & Human Services). The design and results of ACS-QUIK have been reported before (21, 22). The ACS QUIK trial was a pragmatic, cluster randomized, stepped-wedge clinical trial in which hospitals were randomized to receive the quality improvement tool kit intervention at 1 of 5 predefined, 4-month steps over a 24-month period between November 10, 2014, and November 9, 2016, after a period of usual care. The ACS-QUIK study received ethics board approval from local, national, and international bodies and was approved by the Indian Health Ministry Screening Committee. However, among 21,374 patients enrolled in ACS-QUIK the use of quality improvement intervention did not reduce the composite of 30-day major adverse cardiovascular events with either non-ST-segment elevation myocardial infarction (NSTEMI) or ST-segment elevation myocardial infarction (STEMI) in Kerala, India, compared with conventional care. After excluding 815 patients without baseline hemoglobin or outcomes data, we finally enrolled 20,559 patients in our study.

### Exposure Variables and Outcome

The 30-day outcomes were defined from the admission to post-discharge 30-days. The baseline hemoglobin level was defined as the hemoglobin level at the first medical contact. The primary endpoints were 30-day major adverse cardiovascular events defined as death, reinfarction (defined by the Third Universal Definition of Myocardial Infarction) (23), stroke, and major bleeding. The secondary endpoints were 30-day major bleeding [defined by the Global Utilization of Streptokinase and Tissue Plasminogen Activator for Occluded Coronary Arteries (GUSTO) criteria] (24), 30-day stroke and 30-day cardiovascular death.

### Statistical Analysis

We used a normality test to determine whether baseline hemoglobin levels followed a normal distribution. The baseline characteristics of patients were presented as means and standard deviations (SDs) or interquartile ranges (IQRs) for continuous variables depending on whether data distribution was normal [as assessed by normal quantile–quantile (Q–Q) plots and Lilliefors (Kolmogorov-Smirnov) normality test] while categorical variables were expressed as percentages. We compared categorical variables using chi-square tests and continuous variables using one-way analysis of variance (ANOVA) and the Kruskal–Wallis *H*-test, according to the distribution type.

We investigated the relations between baseline hemoglobin level and 30-day adverse outcomes using the baseline hemoglobin level as both continuous and categorical variables by logistic regression model that adjusted clinically meaningful variates and statistically significant covariates. We presented the non-adjusted model, minor adjusted model which adjusted for demographic data, including cohort, intervention, age, male sex and full adjusted models which adjusted for cohort, intervention, age, male sex, ST-segment elevation myocardial infarction (STEMI), heart rate, weight, smoking or tobacco, hypertension, peripheral arterial disease (PAD), prior transient ischemic attack (TIA) or stroke, diabetes, cardiac arrest, Killip class, left-ventricular ejection fraction (LVEF) category, minutes from symptom onset to arrival, angiography, percutaneous coronary intervention (PCI), and inhospital medications. We used multiple imputation, based on five replications and a chained equation approach method in the R multiple imputation procedure, to account for missing data (25). In logistic regression (model 3), we used five sets of data to calculate OR, respectfully, and pooled the results of them. Then we used the generalized additive model (GAM) to determine the non-linear relationship baseline hemoglobin level and 30-day adverse outcomes. When the equivalent degrees of freedom were >1, their relationship was non-linear. If non-linear relationships were identified, we used a two-piecewise linear regression model to calculate the threshold effect of the relationship between baseline hemoglobin level and 30-day adverse outcomes. The recursive method used the maximum-likelihood model to automatically calculate the inflection point with different regression coefficients on the left and right sides of the point, and the ratio of adverse outcomes to baseline hemoglobin level was obvious in the smooth curve. Logarithmic likelihood ratio test was used to compare the differences in associations when using one-line linear regression models vs. two-piecewise linear regression models. We calculated the Akaike Information Criteria (AIC) values of the one-line linear regression models and two-piecewise linear regression models. We used the mean of the five sets of data after imputation to construct the GAM and two-piecewise linear regression models. We preformed the interaction and stratified analyses by all covariates which we included in this study. All analyses were performed with the statistical-software packages R (The R Foundation; http://www.R-project.org) and EmpowerStats (X&Y Solutions, Inc., Boston, Massachusetts, US; http://www.empowerstats.com). *P* < 0.05 (two-sided) were considered statistically significant.

## Results

### Baseline Characteristics of Participants

The baseline characteristics of the ACS-QUIK cohort was report elsewhere (22). In this cohort, the prevalence of anemia [based on the definition provided in 1968 by an expert committee of the World Health Organization (WHO) as a concentration of Hb <13 g/dl in adult men and <12 g/dl in adult non-pregnant women] was 38.1% (7,931) among 20,559 patients with AMI. Baseline hemoglobin levels did not follow a normal distribution [*P* < 0.01, Lilliefors (Kolmogorov-Smirnov) normality test]. At baseline (Table 1), patients with low hemoglobin level were older, more female, had higher heart rate and serum creatinine at admission, and had higher rates of hypertension and diabetes in terms of complications. On the other hand, patients with high baseline hemoglobin level had higher blood pressure and body weight at admission, a higher percentage of smokers, and higher level of triglycerides and low-density lipoprotein cholesterol on laboratory examination. Moreover, for the procedures/treatments of ACS, patients with low hemoglobin levels had longer time from symptom onset to arrival hospital and from door to balloon. In contrast, patients with high hemoglobin level had a higher incidence of STEMI and higher rates of angiography and percutaneous coronary intervention (PCI).

**Table 1:** Baseline characteristics of participants.

**Hemoglobin(g/dL)**	**<10**	**≥10, <11**	**≥11, <12**	**≥12, <13**	**≥13, <14**	**≥14, <15**	**≥15**	***P*-value**
*N*	1,139	1,395	2,431	3,839	4,302	3,796	3,940	
Age	68 (60–75)	67 (59–75)	65 (57–73)	62 (55–70)	60 (52–67)	57 (50–64)	53 (46–60)	<0.001
Sex								<0.001
Female	567 (49.78%)	760 (54.48%)	1,132 (46.57%)	1,278 (33.29%)	853 (19.83%)	351 (9.25%)	123 (3.12%)	
Male	572 (50.22%)	635 (45.52%)	1,299 (53.43%)	2,561 (66.71%)	3,449 (80.17%)	3,445 (90.75%)	3,817 (96.88%)	
Heart rate (BPM)	82 (71–98)	80 (70–92)	78 (68–90)	76 (68–88)	76 (68–88)	76 (68–88)	80 (70–90)	<0.001
SBP (mmHg)	130 (115–160)	130 (110–150)	130 (120–151)	130 (120–150)	135 (120–160)	136 (120–160)	140 (120–160)	<0.001
Weight (Kg)	60 (53–66)	60 (53–66)	60 (55–68)	62 (56–69)	64 (58–70)	65 (59–71)	66 (60–72)	<0.001
CK-MB (units/L)	23.0 (7.0–54.5)	26.0 (8.4–67.0)	29.0 (8.9–77.0)	36.0 (10.0–104.8)	56.1 (12.0–170.0)	51.8 (12.0–101.4)	38.0 (10.5–107.9)	<0.001
Troponin (ng/ml)	1.0 (0.3–4.7)	1.2 (0.3–5.6)	1.2 (0.3–4.9)	1.4 (0.3–5.5)	1.4 (0.3–5.9)	1.3 (0.3–6.1)	1.5 (0.3–6.7)	0.110
Serum creatinine (units/L)	1.3 (1.0–2.0)	1.2 (0.3–5.6)	1.1 (0.9–1.3)	1.0 (0.8–1.2)	1.0 (0.8–1.2)	1.0 (0.9–1.2)	1.0 (0.9–1.2)	<0.001
HDL-C (mg/dL)	40 (34–47)	42 (11) 41 (35–48)	42 (35–49)	41 (35–47)	40 (34–47)	40 (34–47)	41 (35–48)	<0.001
LDL-C(mg/dL)	98 (76–127)	108 (83–137)	114 (88–143)	118 (92–145)	120 (96–146)	124 (99–150)	130 (105–158)	<0.001
TRIG (mg/dL)	101 (76–141)	111 (84–153)	114 (85–153)	119 (89–154)	121 (88–166)	125 (93–171)	135 (97–187)	<0.001
Fasting glucose (mg/dL)	131 (99–185)	130 (102–184)	124 (102–173)	128 (100–174)	128 (101–176)	126 (102–174)	128 (103–174)	0.043
**Risk factors**								
Smoking or tobacco	156 (13.70%)	221 (15.84%)	466 (19.17%)	958 (24.95%)	1,373 (31.92%)	1,460 (38.46%)	1,768 (44.87%)	<0.001
Hypertension	724 (63.56%)	851 (61.00%)	1,315 (54.09%)	1,862 (48.50%)	1,891 (43.96%)	1,568 (41.31%)	1,597 (40.53%)	<0.001
PAD	28 (2.46%)	21 (1.51%)	39 (1.60%)	39 (1.02%)	29 (0.67%)	28 (0.74%)	22 (0.56%)	<0.001
Prior TIA or stroke	52 (4.57%)	58 (4.16%)	62 (2.55%)	84 (2.19%)	87 (2.02%)	46 (1.21%)	68 (1.73%)	<0.001
Diabetes	679 (59.61%)	754 (54.05%)	1,235 (50.80%)	1,762 (45.90%)	1,800 (41.84%)	1,546 (40.73%)	1,505 (38.20%)	<0.001
**Cardiac state**								<0.001
STEMI	497 (43.63%)	671 (48.10%)	1,395 (57.38%)	2,345 (61.08%)	2,879 (66.92%)	2,663 (70.15%)	2,819 (71.55%)	
Heart failure	152 (13.35%)	182 (13.05%)	237 (9.75%)	365 (9.51%)	310 (7.21%)	296 (7.80%)	332 (8.43%)	<0.001
Cardiac shock	46 (4.04%)	51 (3.66%)	68 (2.80%)	103 (2.68%)	80 (1.86%)	68 (1.79%)	92 (2.34%)	<0.001
Cardiac arrest	22 (1.93%)	18 (1.29%)	34 (1.40%)	51 (1.33%)	50 (1.16%)	44 (1.16%)	72 (1.83%)	0.082
**Killip class**								<0.001
1	864 (75.86%)	1,107 (79.35%)	2,056 (84.57%)	3,316 (86.38%)	3,837 (89.19%)	3,415 (89.96%)	3,441 (87.36%)	
2	75 (6.58%)	86 (6.16%)	138 (5.68%)	217 (5.65%)	194 (4.51%)	185 (4.87%)	253 (6.42%)	
3	158 (13.87%)	151 (10.82%)	178 (7.32%)	207 (5.39%)	196 (4.56%)	134 (3.53%)	182 (4.62%)	
4	42 (3.69%)	51 (3.66%)	59 (2.43%)	99 (2.58%)	75 (1.74%)	62 (1.63%)	63 (1.60%)	
**LVEF category**								<0.001
1	215 (18.88%)	262 (18.78%)	369 (15.18%)	530 (13.81%)	525 (12.20%)	428 (11.28%)	529 (13.43%)	
2	757 (66.46%)	875 (62.72%)	1,626 (66.89%)	2,689 (70.04%)	3,033 (70.50%)	2,751 (72.47%)	2,783 (70.63%)	
3	39 (3.42%)	71 (5.09%)	115 (4.73%)	189 (4.92%)	249 (5.79%)	215 (5.66%)	240 (6.09%)	
4	128 (11.24%)	187 (13.41%)	321 (13.20%)	431 (11.23%)	495 (11.51%)	402 (10.59%)	388 (9.85%)	
**Procedures**								
Symptom onset to arrival(min)	340 (135–1,108)	298 (127–990)	270 (120–894)	255 (120–870)	220 (111–798)	225 (107–760)	246 (120–750)	<0.001
Angiography	411 (36.08%)	577 (41.36%)	1,219 (50.14%)	2,204 (57.41%)	2,711 (63.02%)	2,502 (65.91%)	2,822 (71.62%)	
PCI	274 (24.06%)	422 (30.25%)	969 (39.86%)	1,746 (45.48%)	2,360 (54.86%)	2,267 (59.72%)	2,326 (59.04%)	<0.001
CABG	4 (0.35%)	2 (0.14%)	18 (0.74%)	22 (0.57%)	18 (0.42%)	23 (0.61%)	20 (0.51%)	0.207
Door to balloon(min)	85 (55–206)	92 (60–232)	85 (55–220)	80 (55–205)	80 (60–195)	80 (60–178)	84 (55–185)	0.036
**Meditation**								
Aspirin	930 (92.54%)	1,183 (94.79%)	2,154 (97.11%)	3,425 (97.69%)	3,893 (98.09%)	3,503 (98.26%)	3,647 (98.43%)	<0.001
Clopidogrel	895 (89.05%)	1,104 (88.46%)	1,982 (89.36%)	3,070 (87.56%)	3,333 (83.98%)	2,901 (81.37%)	2,900 (78.27%)	<0.001
Ticagrelor	51 (5.07%)	100 (8.01%)	169 (7.62%)	306 (8.73%)	431 (10.86%)	438 (12.29%)	460 (12.42%)	<0.001
Beta blocker	563 (56.02%)	724 (58.01%)	1,321 (59.56%)	2,130 (60.75%)	2,626 (66.16%)	2,453 (68.81%)	2,540 (68.56%)	<0.001
Warfarin	18 (1.79%)	22 (1.76%)	43 (1.94%)	90 (2.57%)	68 (1.71%)	83 (2.33%)	55 (1.48%)	0.174
ACEI	279 (27.76%)	420 (33.65%)	795 (35.84%)	1,383 (39.45%)	1,628 (41.02%)	1,554 (43.59%)	1,521 (41.05%)	<0.001
ARB	116 (11.54%)	125 (10.02%)	204 (9.20%)	308 (8.78%)	354 (8.92%)	282 (7.91%)	344 (9.28%)	<0.001
Statin	961 (95.62%)	1,203 (96.39%)	2,144 (96.66%)	3,389 (96.66%)	3,862 (97.30%)	3,448 (96.72%)	3,589 (96.87%)	0.427
**Outcomes**								
Major bleeding	9 (0.80%)	5 (0.37%)	11 (0.46%)	5 (0.13%)	3 (0.07%)	8 (0.21%)	4 (0.10%)	<0.001
Stroke	9 (0.80%)	14 (1.02%)	27 (1.13%)	24 (0.64%)	22 (0.52%)	17 (0.45%)	26 (0.67%)	0.025
CVD death	109 (9.67%)	116 (8.49%)	124 (5.18%)	158 (4.19%)	144 (3.39%)	86 (2.29%)	87 (2.24%)	<0.001
MACE	131 (11.62%)	147 (10.74%)	164 (6.84%)	230 (6.09%)	182 (4.28%)	139 (3.70%)	142 (3.66%)	<0.001

*SBP, systolic blood pressure; BPM, beats per minute; CK-MB, creatine kinase isoenzymes in the heart; Hb, hemoglobin; HDL-C, high-density lipoprotein cholesterol; LDL-C, low-density lipoprotein cholesterol; TRIG, triglycerides; PAD, peripheral arterial disease; TIA, transient ischemic attack; STEMI, ST-segment elevation myocardial infarction; PCI, percutaneous coronary intervention; LVEF, left-ventricular ejection fraction; LVEF category, 1: ≤40%; 2: 40–70%; 3: ≥70%; 4, unknown or not assessed; ACEI, angiotensin-converting enzyme inhibitor; ARB, angiotensin receptor blocker; CABG, coronary-artery bypass graft surgery; MACE, major adverse cardiovascular events*.

*P was calculated by 1-way analysis of variance (ANOVA) and the Kruskal–Wallis H-test, according to distribution type*.

### Relationship Between Baseline Hemoglobin Level and Adverse Outcomes

We presented the non-adjusted model, minor adjusted model, and full adjusted model in the Table 2. In the non-adjusted model, as hemoglobin levels increased, the risk of adverse events decreased [Major bleeding, OR: 0.73, 95%CI (0.64, 0.83), *P* < 0.01, Stroke, OR: 0.92, 95%CI (0.85, 1.00), *P* = 0.04, CVD death, OR: 0.79, 95%CI (0.77, 0.82), *P* < 0.01, MACE, OR: 0.82, 95%CI (0.80, 0.85), *P* < 0.01]. However, in the full adjusted model baseline hemoglobin level was not statistically significant in relation to Stroke [OR: 1.03, 95%CI (0.93, 1.13), *P* = 0.61].

**Table 2:** Relationship between baseline hemoglobin level and adverse outcomes in different models.

**Outcomes**	**Non-adjusted**	**Adjusted I**	**Adjusted II**
Major bleeding	0.73 (0.64, 0.83), *P* < 0.01, *n* = 20,554	0.74 (0.64, 0.85), *P* < 0.01, *n* = 20,554	0.76 (0.65, 0.89), *P* < 0.01, *n* = 20,554
Stroke	0.92 (0.85, 1.00), *P* = 0.04, *n* = 20,556	1.00 (0.91, 1.10), *P* = 0.95, *n* = 20,556	1.03 (0.93, 1.13), *P* = 0.61, *n* = 20,556
CVD death	0.79 (0.77, 0.82), *P* < 0.01, *n* = 20,553	0.89 (0.86, 0.93), *P* < 0.01, *n* = 20,553	0.94 (0.90, 0.98), *P* < 0.01, *n* = 20,553
MACEs	0.82 (0.80, 0.85), *P* < 0.01, *n* = 20,559	0.91 (0.88, 0.94), *P* < 0.01, *n* = 20,559	0.95 (0.92, 0.99), *P* < 0.01, *n* = 20,559

*MACE, major adverse cardiovascular events. In the Adjusted I model, we adjusted demographic data, including cohort, intervention, age, and male sex. In the Adjusted II model, we adjusted all confounders, including cohort, intervention, age, male sex, ST-segment elevation myocardial infarction (STEMI), heart rate, weight, smoking or tobacco, hypertension, peripheral arterial disease (PAD), prior transient ischemic attack (TIA) or stroke, diabetes, cardiac arrest, Killip class, left-ventricular ejection fraction (LVEF) category, minutes from symptom onset to arrival, angiography, percutaneous coronary intervention (PCI), prescribed aspirin, prescribed clopidogrel, prescribed ticagrelor, prescribed beta blocker, prescribed warfarin, prescribed angiotensin-converting enzyme inhibitor (ACEI), prescribed statin*.

### Analyses of Non-linear Relationship

**Figure 1:**
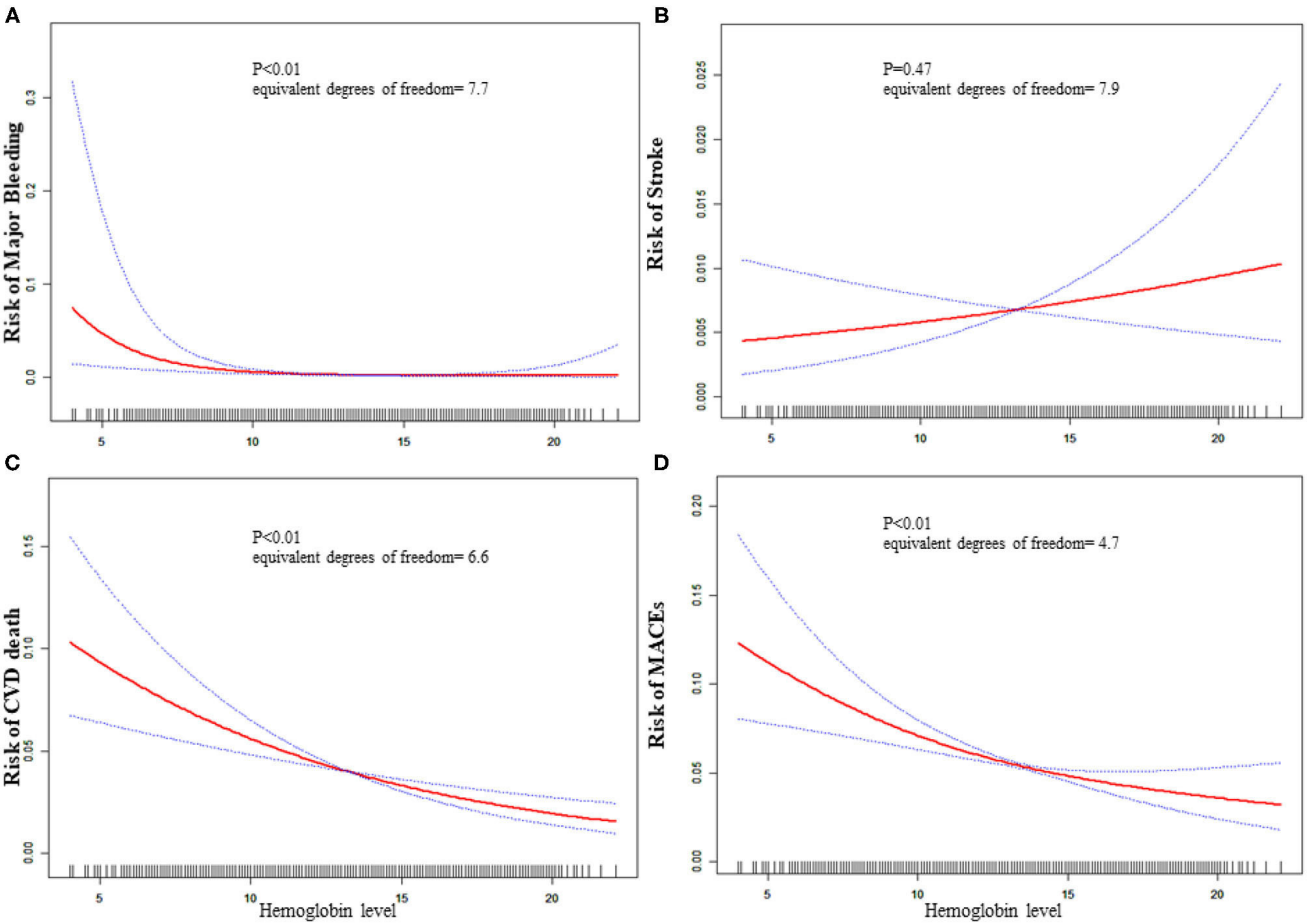
Relationship between baseline hemoglobin level and 30-day adverse outcomes. **(A)** Major bleeding, **(B)** stroke, **(C)** CVD death, **(D)** MACE. The red line is the trend line and the blue line is the 95% confidence interval. The denser the vertical lines, the greater the number of patients in the area.

The equivalent degrees of freedom of GAM were 7.7, 6.6, and 4.6 for major bleeding, CVD death, and MACEs, respectively. Generalized additive model showed non-linear relationships between baseline hemoglobin level and major bleeding, CVD death, and MACEs (Figure 1). Then we used two-piecewise linear regression models (Table 3), and we found the threshold value was 13, 14.8, and 14.3 g/dL for major bleeding, CVD death, and MACEs, respectively. In those two-piecewise linear regression models of major bleeding, CVD death, and MACEs, the AIC values were less than the one-line linear regression models (Supplementary Table 1). Above the threshold point, baseline hemoglobin level did not associate with major bleeding, CVD death, nor MACE. However, below the threshold point, the increase in baseline hemoglobin level was associated with a lower risk of major bleeding [OR: 0.65, 95%CI (0.53, 0.79), *P* < 0.01], CVD death [OR: 0.87, 95%CI (0.82, 0.92), *P* < 0.01], and MACE [OR: 0.89, 95%CI (0.85, 0.93), *P* < 0.01].

**Table 3:** Results of two-piecewise linear-regression model.

	**Female**	**Male**	**Total**
**Major bleeding**	***N* = 4,981**	***N* = 15,569**	***N* = 20,550**
One linear-regression model	0.89 (0.56, 1.41) *P* = 0.61	0.71 (0.58, 0.85) *P* < 0.01	0.76 (0.65, 0.89), *P* < 0.01
Inflection point (K)	10	13	13
< K Effect size β (95%CI)	3.32 (0.08, 130.77) *P* = 0.52	0.57 (0.44, 0.72) *P* < 0.01	0.65 (0.53, 0.79) *P* < 0.01
>K Effect size β (95%CI)	0.71(0.36, 1.40) *P* = 0.32	1.14 (0.77, 1.66) *P* = 0.52	1.06 (0.75, 1.50) *P* = 0.74
*P* for Log likelihood ratio test	0.30	0.02	0.05
**Stroke**	***N*** **= 4,982**	***N*** **= 15,570**	***N*** **= 20,552**
One linear-regression model	1.15 (0.96, 1.37) *P* = 0.18	1.02 (0.90, 1.14) *P* = 0.77	1.03 (0.93, 1.13) *P* = 0.61
Inflection point (K)	10.5	16.5	10.4
< K Effect size β (95%CI)	1.86 (0.89, 3.86) *P* = 0.10	1.04 (0.92, 1.18) *P* = 0.53	1.34 (0.86, 2.08) *P* = 0.19
>K Effect size β (95%CI)	1.01 (0.78, 1.30) *P* = 0.94	0.54 (0.13, 2.19) *P* = 0.39	1.01 (0.89, 1.13) *P* = 0.93
*P* for Log likelihood ratio test	0.13	0.29	0.22
**CVD death**	***N*** **= 4,981**	***N*** **= 15,568**	***N*** **= 20,549**
One linear-regression model	0.89 (0.80, 0.98) *P* < 0.01	0.89 (0.84, 0.95) *P* < 0.01	0.94 (0.90, 0.98) *P* < 0.01
Inflection point (K)	9.2	14.9	14.8
< K Effect size β (95%CI)	1.31 (0.85, 2.01) *P* = 0.22	0.85 (0.80, 0.92) *P* < 0.01	0.87 (0.82, 0.92) *P* < 0.01
>K Effect size β (95%CI)	0.83 (0.74, 0.94) *P* < 0.01	1.15 (0.93, 1.43) *P* = 0.20	1.17 (0.96, 1.42) *P* = 0.13
*P* for Log likelihood ratio test	0.06	0.02	<0.01
**Mace**	***N*** **= 4,983**	***N*** **= 15,572**	***N*** **= 20,555**
One linear-regression model	0.95(0.87, 1.02) *P* = 0.16	0.91 (0.87, 0.95) *P* < 0.01	0.95 (0.91, 0.98) *P* = 0.01
Inflection point (K)	9.2	13.8	14.3
< K Effect size OR (95%CI)	1.26 (0.86, 1.84) *P* = 0.24	0.86 (0.80, 0.92) *P* < 0.01	0.89 (0.85, 0.93) *P* < 0.01
>K Effect size β (95%CI)	0.91 (0.82, 1.00) *P* = 0.04	1.02 (0.92, 1.14) *P* = 0.69	1.07 (0.95, 1.21) *P* = 0.26
*P* for Log likelihood ratio test	0.11	0.02	<0.01

*MACE, major adverse cardiovascular events; CVD death, cardiovascular death. We adjusted for cohort, intervention, age, male sex, ST-segment elevation myocardial infarction (STEMI), heart rate, weight, smoking or tobacco, hypertension, peripheral arterial disease (PAD), prior transient ischemic attack (TIA) or stroke, diabetes, cardiac arrest, cardiac shock, heart failure, Killip class, left-ventricular ejection fraction (LVEF) category, minutes from symptom onset to arrival, angiography, percutaneous coronary intervention (PCI), prescribed aspirin, prescribed clopidogrel, prescribed prasugrel, prescribed ticagrelor, prescribed beta blocker, prescribed warfarin, prescribed angiotensin-converting enzyme inhibitor (ACEI), prescribed statin*.

*The 2-piecewise linear regression model was used to calculate the threshold effect*.

### Sex Difference in the Association With the Baseline Hemoglobin Level and Adverse Outcomes

**Figure 2:**
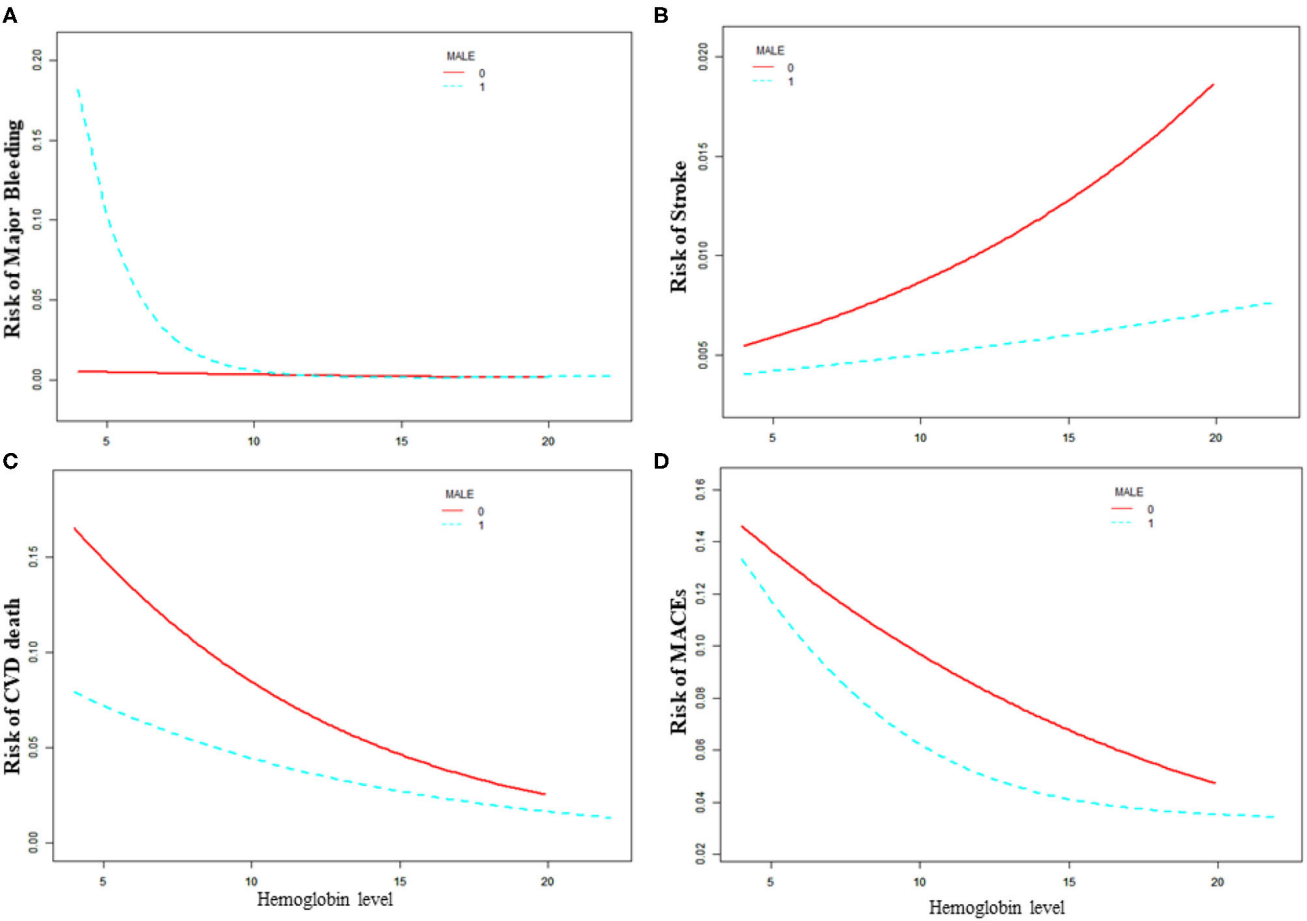
Relationship between baseline hemoglobin level and 30-day adverse outcomes grouped by sex. **(A)** Major bleeding, **(B)** stroke, **(C)** CVD death, **(D)** MACE. Male 0: female, Male 1: male.

As is shown in Table 3 and Figure 2, the increased risk of major bleeding from low hemoglobin level was mainly concentrated in the male population [OR: 0.71, 95%CI (0.58, 0.85) *P* < 0.01], but not in the female population [OR: 0.89, 95%CI (0.56, 1.41) *P* = 0.61]. However, in female patients, the risk of stroke increased as hemoglobin increased, but it was not statistically significant [OR: 1.15, 95%CI (0.96, 1.37) *P* = 0.18]. The risk of stroke in male patients did not change with changing of hemoglobin level [OR: 1.02, 95%CI (0.90, 1.14) *P* = 0.77]. The risk of CVD death was significantly reduced when the hemoglobin level was below 14.9 g/dL as hemoglobin level increased [OR: 0.85, 95%CI (0.80, 0.92) *P* < 0.01] of the male patients. On the other hand, the risk of CVD death of female patients lowered [OR: 0.83, 95%CI (0.74, 0.94) *P* < 0.01] when the hemoglobin level was above 9.2 g/dL. As the level of hemoglobin increased before the inflection point, the risk of MACE in men was significantly reduced [OR: 0.86, 95%CI (0.80, 0.92) *P* < 0.01], while women had no significant impact [OR: 1.26, 95%CI (0.86, 1.84) *P* = 0.24].

### Sensitivity Analysis

As showed in Supplementary Table 2, for major bleeding, the statistically significant variables of the interaction test were as follows: age, weight, smoking or tobacco, Killip class, Clopidogrel, and Beta blocker (*P* for interaction is 0.03, <0.01, <0.01, <0.01, <0.01, and 0.02). For stroke, the significant variables of the interaction test were weight and LVEF category (*P* for interaction is 0.02 and 0.02). We observed that high hemoglobin levels were a risk factor for stroke in low-weight populations [OR: 1.20, 95%CI (1.01, 1.43) *P* = 0.04], while high hemoglobin was a protective factor for stroke in high-weight populations [OR: 0.76, 95%CI (0.57, 1.01) *P* = 0.06]. For MACE, the significant variables of the interaction test were heart failure (*P* for interaction is 0.05).

As showed in Supplementary Tables 3–5, we used the data before multiple imputation to conduct sensitivity analysis and compared it with the results after imputation, which did not have a significant impact on the results.

## Discussion

Anemia was associated with multiple comorbidities, such as diabetes (26), heart failure (27, 28), chronic kidney disease (29), and other non-cardiovascular conditions (30, 31). Although many studies confirmed that anemia was an independent predictor of prognosis in patients with AMI (10, 32–34), few studies explored the impact of hemoglobin levels on the prognosis of patients with AMI (35–37).

Brener et al. only described the non-linear relationship between baseline hemoglobin level and prognosis, but their study did not elaborate on the independent effects of different hemoglobin levels on prognosis (20). The main findings of this study, based on an analysis of more than 20,000 patients with AMI in India, were as follows: (1) There was a non-linear relationship between baseline hemoglobin levels and adverse events, and there was a threshold. We obtained the inflection point by the maximum likelihood method. (2) We found a sex difference in the relationship between baseline hemoglobin levels and adverse outcomes by interaction test. (3) This was the first study to determine the relationship between the baseline hemoglobin level with adverse outcomes in South Asia where had the highest burden of anemia.

The relationship between baseline hemoglobin level and adverse outcomes approximated a J-shaped curve (5), and we found the lowest incidence of adverse events when patients' hemoglobin level was about 14–15 g/dl. In the Organization to Assess Strategies in Acute Ischemic Syndromes (OASIS) 5 and 6 trials, Bassand et al. reported that the hazard for death seemed to be lowest at a hemoglobin level of 15.9 g/dl (19). Sabatine et al. included more than 40,000 ACS patients and calculated that the lowest risk was between 14–15 g/dl for STEMI and 15–16 g/dl for NSTEMI (1). And their hemoglobin value was greater than the value (14.3 g/dl) in our study.

Different levels of hemoglobin had different effects on the cardiovascular system. Low hemoglobin levels mean a reduction in the ability of the blood to carry oxygen. The delivery of oxygen depended primarily on hemoglobin and cardiac output. In the condition of AMI, the systolic and diastolic functions of ventricles were affected by local myocardial hypoperfusion due to the involvement of the coronary arteries, resulting in decreased cardiac output. Conversely, if tissue oxygen demand was insufficient, heart rate increased faster to increase cardiac output, which would increase cardiac workload and might exacerbate myocardial supply and demand mismatch. Additionally, high hemoglobin levels might directly contribute to the formation of acute thrombosis and increase the risk of thrombosis in patients with polycythemia (38). At the same time, high hemoglobin levels could lead to vascular endothelial damage and rupture of vulnerable plaque (39–42). Recent studies showed that reduced hemoglobin was associated with an increased risk of adverse events in ACS patients, even if they did not have bleeding events. This conclusion indicated that ACS patients with reduced hemoglobin levels should receive more attention and correct anemia, regardless of the patient's bleeding events (12).

Low hemoglobin levels could also lead to an increased risk of bleeding. A low baseline hemoglobin level might be a sign of occult gastrointestinal bleeding, inflammatory status, or hemorrhagic quality. In addition, hematocrit levels could affect primary hemostasis. It was shown that an increase in the hematocrit level on the one hand could lead to an increase in platelet deposition on the arterial wall, and on the other hand led to an increase in blood viscosity and an increase in shear force. In this case, the function of platelets might be activated by adenosine diphosphate released by red blood cells (43, 44).

Many studies reported that low hemoglobin levels were associated with bleeding and the women have a higher bleeding risk in ACS (19, 44). However, in our study, the increased risk of bleeding caused by low hemoglobin level was more pronounced in male patients. In female patients, the level of hemoglobin was not significantly related to the risk of bleeding. Differences in population race, sex and age might lead to differences in the relationship between hemoglobin and adverse outcomes, which was reported in previous studies (45). Based on the results of this study, we should be aware of the risk of bleeding when treating male patients with low hemoglobin levels and should consider a more conservative treatment strategy in antiplatelet therapy.

## Limitation

There were still many limitations in our research. Although we adjusted a lot of variables, but limited to the original database, we were unable to adjust all the variables that need to be adjusted. Several variables in the Global Registry of Acute Coronary Events (GRACE) score, such as excessive deletion of creatine kinase isoenzymes in the heart, ST segment depression, were not included. Moreover, the raw data did not collect the cause of patients' hemoglobin declines. Therefore, we did not know whether the reason for the decrease in hemoglobin levels in patients was related to adverse outcomes. There were 1,741 patients with no medication data, and although we used the method of multiple imputation, we were not able to completely avoid this possible bias. This study was a *post-hoc* analysis of a pre-defined registry with a study population from the south Asia. The results of this study might not be applicable to Western countries due to differences in the ethnicity and treatment of AMI patients.

## Conclusion

The level of baseline hemoglobin was an independent predictor of prognosis in patients with acute myocardial infarction in South Asia. Moreover, its effect on prognosis was largely dependent on the sex of the patient. Low hemoglobin levels could increase the risk of adverse outcomes in patients with acute myocardial infarction. Male patients with low hemoglobin levels were at high risk of bleeding and should consider conservative antiplatelet and anticoagulation strategies.

## Data Availability Statement

Publicly available datasets were analyzed in this study. This data can be found here: https://biolincc.nhlbi.nih.gov/studies/acs_quik/.

## Author Contributions

XH and XW designed the study and provided methodological expertise. JP and XW drafted the manuscript. XW, JP, PC, KZ, and XH drafted the tables and figures and performed statistical analysis. JP was mainly responsible for the revised manuscript. All authors have read and approved the final manuscript.

## Conflict of Interest

The authors declare that the research was conducted in the absence of any commercial or financial relationships that could be construed as a potential conflict of interest.
