# Supplementary material for: Hb Levels and Sex Differences in Relation to Short-Term Outcomes in Patients With Acute Myocardial Infarction
Source: Front Cardiovasc Med. 2021 Jul 16;8:653351. doi: 10.3389/fcvm.2021.653351 (PMC8322114; doi:10.3389/fcvm.2021.653351)
Supplement: Supplementary file 1 [file Table_1.docx]

**
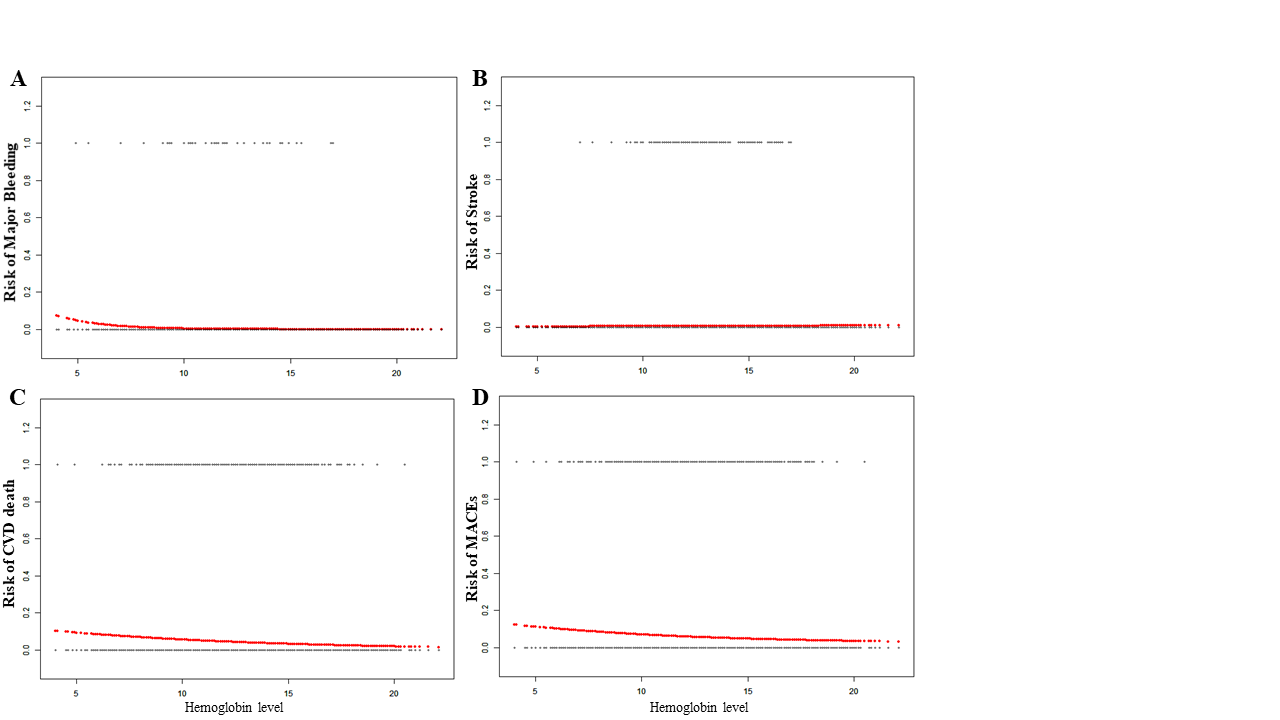
**

**Supplemental Figure 1.** The scatter plot of GAM of the all patients. Every dot representing individual patients.

**
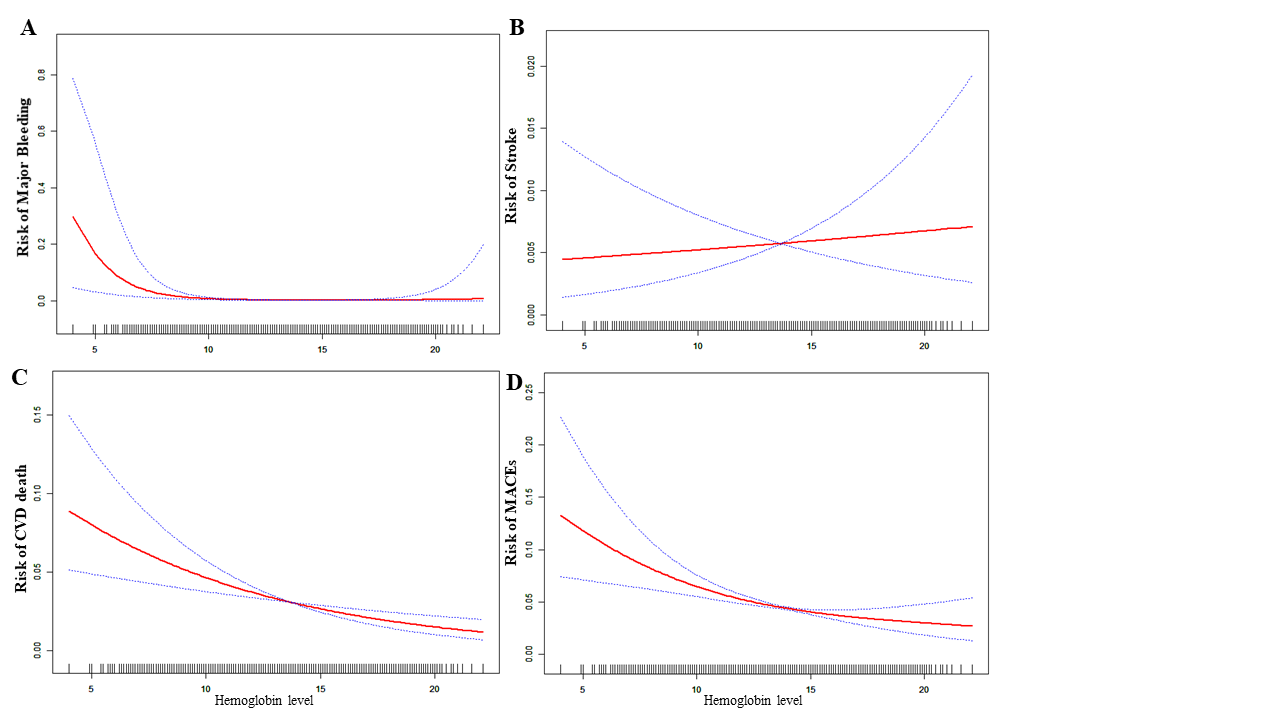
**

**Supplemental Figure 2.** Relationship between baseline hemoglobin level and 30-day adverse outcomes in male patients. A: Major bleeding, B: Stroke, C: CVD death, D: MACE. The red line is the trend line and the blue line is the 95% confidence interval. The denser the vertical lines, the greater the number of patients in the area.

**
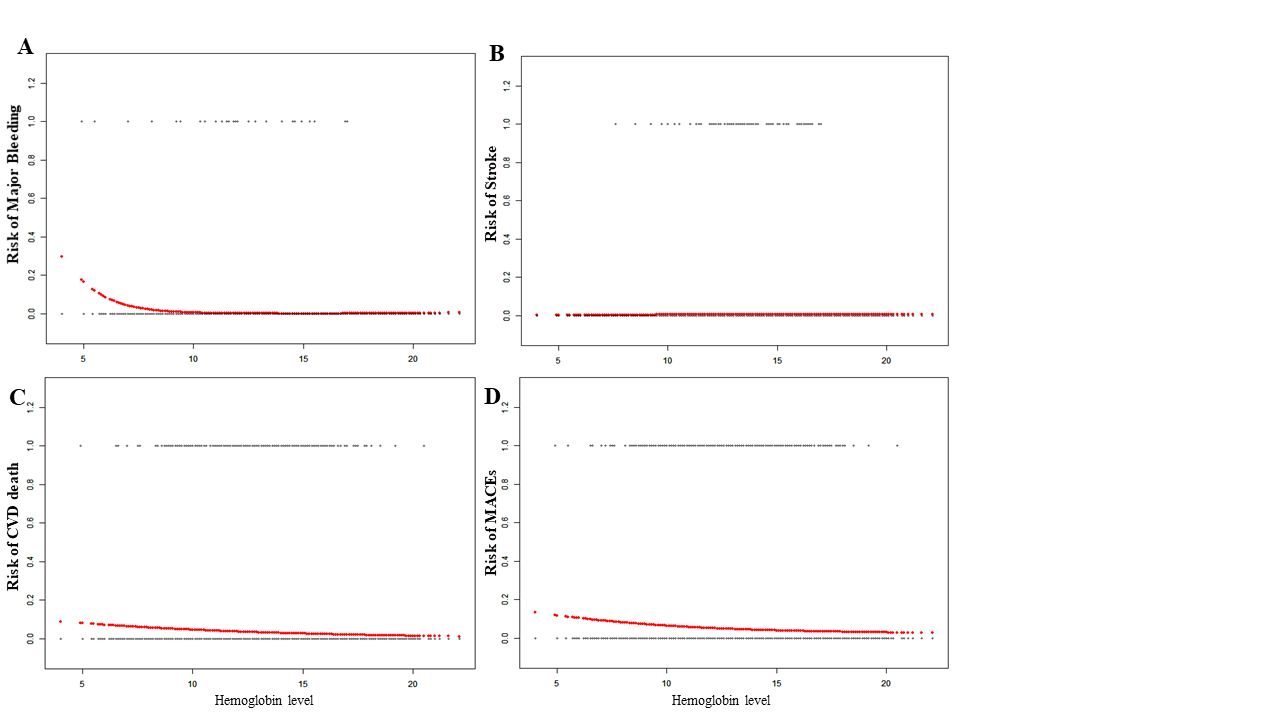
**

**Supplemental Figure 3.** The scatter plot of GAM of the male patients. Every dot representing individual patients.

**
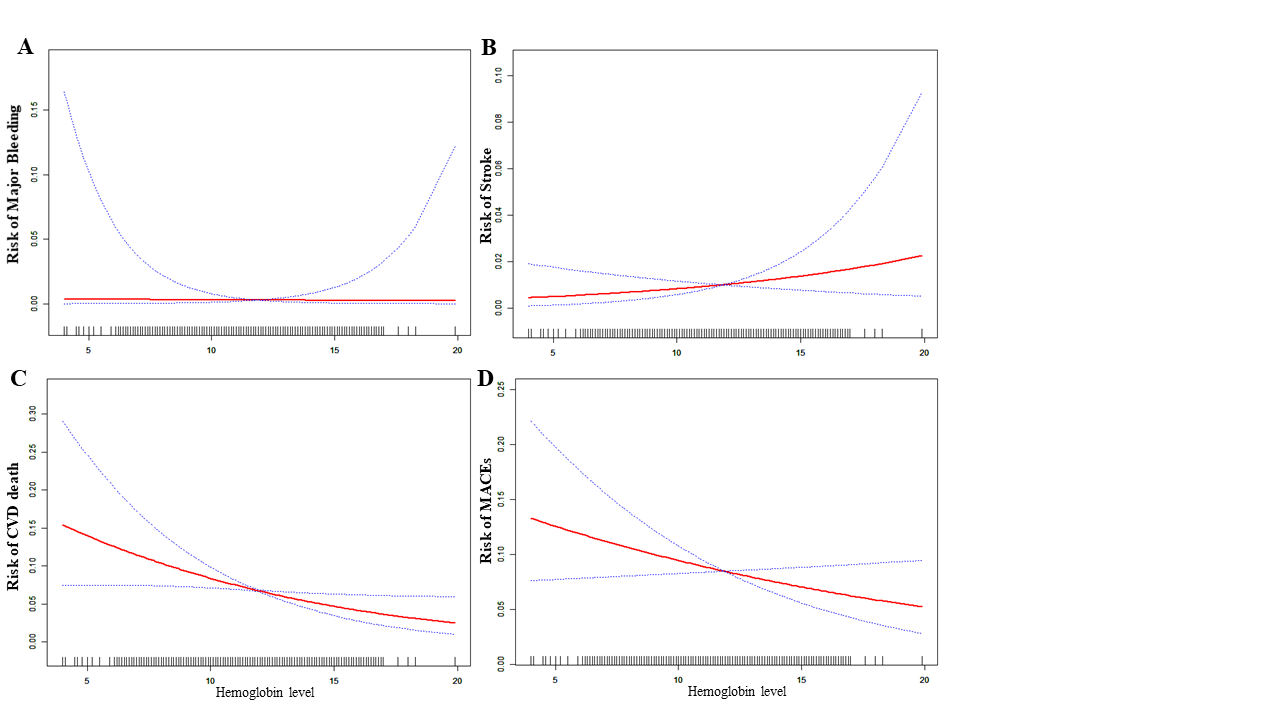
**

**Supplemental Figure 4.** Relationship between baseline hemoglobin level and 30-day adverse outcomes in female patients. A: Major bleeding, B: Stroke, C: CVD death, D: MACE. The red line is the trend line and the blue line is the 95% confidence interval. The denser the vertical lines, the greater the number of patients in the area.

**
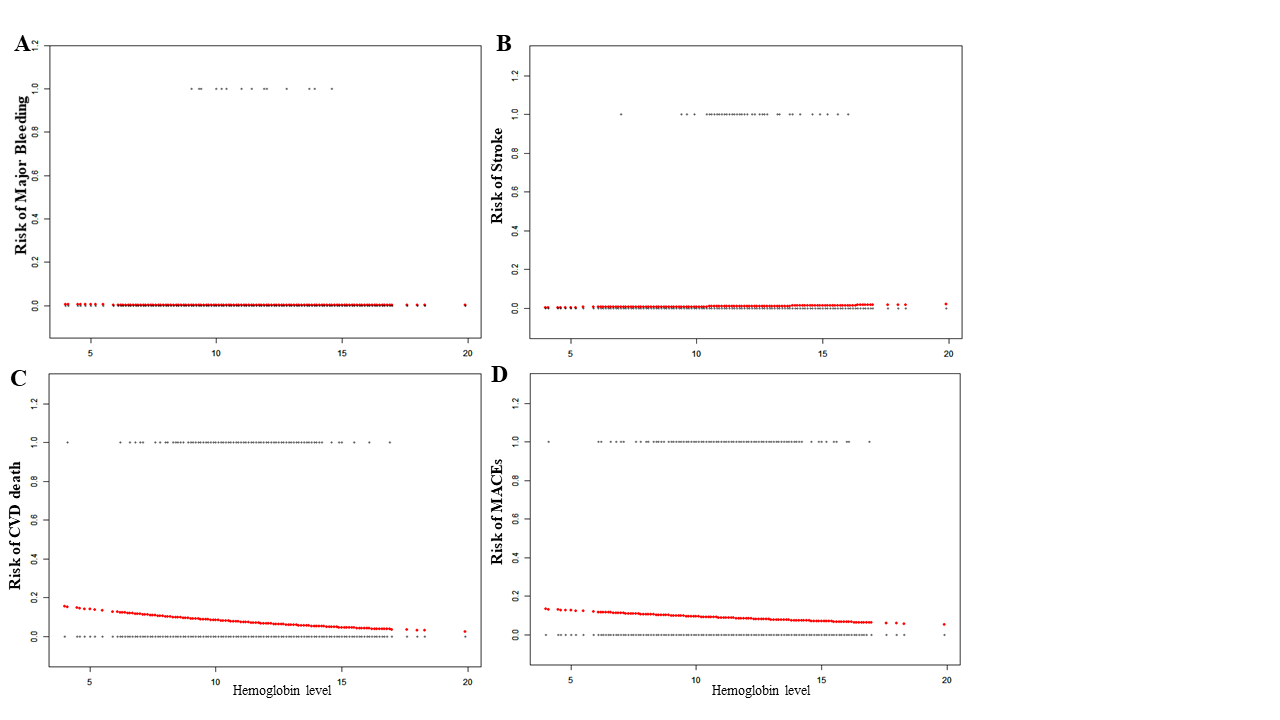
**

**Supplemental Figure 5.** The scatter plot of GAM of the female patients. Every dot representing individual patients.

**Supplemental Table 1. The Akaike Information Criteria (AIC) values of the one-line linear regression models and two-piecewise linear regression models.**

|  | AIC values of one-line linear regression model | AIC values of two-piecewise linear regression model |
| --- | --- | --- |
| All participants |  |  |
| Major bleeding | -67607 | -67622 |
| Stroke | -44828 | -44826 |
| CVD death | -10616 | -10626 |
| MACEs | -4263.1 | -4274.6 |
| Male |  |  |
| Major bleeding | -52708 | -52841 |
| Stroke | -36665 | -36663 |
| CVD death | -11483 | -11490 |
| MACEs | -5739.9 | -5751.4 |
| Female |  |  |
| Major bleeding | -15112 | -15109 |
| Stroke | -8853.1 | -8852 |
| CVD death | -266.96 | -264.94 |
| MACEs | 821.03 | 822.81 |

**Supplemental Table 2. The interaction test and subgroup analysis.**

|  | Major bleeding | | | Stroke | | | CVD death | | | MACE | | |
| --- | --- | --- | --- | --- | --- | --- | --- | --- | --- | --- | --- | --- |
|  | OR (95% CI) | *P*-value | *P* interaction | OR (95% CI) | *P*-value | *P* interaction | OR (95% CI) | *P*-value | *P* interaction | OR (95% CI) | *P*-value | *P* interaction |
| Age |  |  | 0.03 |  |  | 0.4942 |  |  | 0.2163 |  |  | 0.0424 |
| Young | 1.75(0.76,4.04) | 0.1872 | N=6324 | 0.98(0.76,1.25) | 0.8415 | N=6324 | 1.01(0.84,1.23) | 0.8826 | N=6323 | 1.05(0.95,1.18) | 0.3359 | N=6326 |
| Middle | 0.52(0.33,0.81) | 0.0037 | N=6045 | 1.20(0.81,1.58) | 0.1879 | N=6045 | 0.84(0.72,0.98) | 0.0275 | N=6045 | 0.88(0.79,0.97) | 0.0082 | N=6045 |
| Old | 0.67(0.46,0.99) | 0.0417 | N=6619 | 1.02(0.87,1.20) | 0.8004 | N=6619 | 0.97(0.88,1.07) | 0.5875 | N=6619 | 0.97(0.91,1.04) | 0.4561 | N=6619 |
| Heart rate |  |  | 0.1176 |  |  | 0.6855 |  |  | 0.5684 |  |  | 0.0552 |
| Low | 0.37(0.14,0.99) | 0.0486 | N=6360 | 1.09(0.85,1.41) | 0.5076 | N=6360 | 0.88(0.75,1.41) | 0.1449 | N=6360 | 0.90(0.81,1.01) | 0.0648 | N=6361 |
| Moderate | 1.10(0.60,2.02) | 0.7522 | N=6610 | 1.10(0.87,1.40) | 0.4316 | N=6110 | 0.99(0.86,1.13) | 0.8528 | N=6109 | 1.06(0.96,1.16) | 0.24 | N=6110 |
| High | 0.66(0.49,0.91) | 0.01 | N=6518 | 0.98(0.83,1.16) | 0.857 | N=6518 | 0.97(0.87,1.08) | 0.578 | N=6518 | 0.94(0.87,1.01) | 0.0709 | N=6519 |
| Systolic blood pressure |  |  | 0.0877 |  |  | 0.6381 |  |  | 0.3408 |  |  | 0.4753 |
| Low | 0.44(0.29,0.66) | <0.0001 N=6129 | | 1.05(0.86,1.29) | 0.6404 | N=6129 | 0.92(0.81,1.04) | 0.1616 | N=6129 | 0.92(0.84,1.00) | 0.0537 | N=6131 |
| Moderate | None |  | N=5966 | 0.93(0.73,1.18) | 0.5509 | N=5966 | 0.93(0.82,1.06) | 0.2718 | N=5966 | 0.96(0.87,1.05) | 0.6308 | N=5966 |
| High | 0.82(0.55,1.20) | 0.3003 | N=6893 | 1.07(0.89,1.29) | 0.4272 | N=6893 | 1.01(0.87,1.16) | 0.9338 | N=6892 | 0.99(0.91,1.08) | 0.8456 | N=6893 |
| Weight |  |  | 0.0034 |  |  | 0.024 |  |  | 0.3583 |  |  | 0.2861 |
| Low | 0.73(0.51,1.04) | 0.0839 | N=6112 | 1.20(1.01,1.43) | 0.0371 | N=6112 | 0.99(0.89,1.11) | 0.8904 | N=6112 | 1.00(0.93,1.09) | 0.9202 | N=6113 |
| Moderate | 0.23(0.08,0.67) | 0.0071 | N=6183 | 1.00(0.81,1.24) | 0.9755 | N=6183 | 0.89(0.78,1.01) | 0.0705 | N=6183 | 0.91(0.84,1.00) | 0.0419 | N=6184 |
| High | 1.31(0.72,2.39) | 0.374 | N=6693 | 0.76(0.57,1.01) | 0.0571 | N=6693 | 1.00(0.86,1.18) | 0.9567 | N=6692 | 0.97(0.88,1.08) | 0.5826 | N=6693 |
| Smoking or tobacco |  |  | <0.0001 |  |  | 0.8491 |  |  | 0.2467 |  |  | 0.1564 |
| No | 0.78(0.61,1.01) | 0.057 | N=13269 | 1.03(0.90,1.19) | 0.649 | N=13269 | 0.98(0.90,1.07) | 0.6766 | N=13268 | 0.99(0.93,1.05) | 0.6332 | N=13270 |
| Yes | None |  | N=5719 | 1.06(0.85,1.32) | 0.602 | N=5719 | 0.89(0.77,1.03) | 0.1086 | N=5719 | 0.91(0.83,1.00) | 0.0458 | N=5720 |
| Hypertension |  |  | 0.5179 |  |  | 0.1863 |  |  | 0.4314 |  |  | 0.2753 |
| No | 0.64(0.41,0.99) | 0.0454 | N=10025 | 1.13(0.94,1.36) | 0.1961 | N=10025 | 0.92(0.82,1.03) | 0.1294 | N=10024 | 0.93(0.86,1.00) | 0.0593 | N=10026 |
| Yes | 0.76(0.57,1.01) | 0.0542 | N=8693 | 0.96(0.82,1.12) | 0.6068 | N=8693 | 0.97(0.88,1.08) | 0.6167 | N=8963 | 0.98(0.92,1.05) | 0.6144 | N=8964 |
| PAD |  |  | None |  |  | None |  |  | None |  |  | 0.2809 |
| No | 0.74(0.60,0.92) | 0.0059 | N=18809 | 1.04(0.92,1.17) | 0.5297 | N=18809 | 0.95(0.88,1.02) | 0.1885 | N=18808 | 0.86(0.91,1.01) | 0.0896 | N=18811 |
| Yes | None |  | N=179 | None |  | N=179 | None |  | N=179 | 1.42(0.68,2.98) | 0.3551 | N=179 |
| Prior stroke or TIA |  |  | None |  |  | 0.6084 |  |  | 0.7286 |  |  | 0.5838 |
| No | 0.74(0.60,0.92) | 0.0059 | N=18595 | 1.01(0.89,1.14) | 0.5932 | N=18595 | 0.95(0.88,1.03) | 0.2412 | N=18594 | 0.96(0.91,1.01) | 0.0962 | N=18596 |
| Yes | None |  | N=393 | 1.13(0.75,1.68) | 0.5644 | N=383 | 0.90(0.64,1.26) | 0.5357 | N=393 | 1.03(0.79,1.34) | 0.822 | N=394 |
| Diabetes |  |  | 0.989 |  |  | 0.5755 |  |  | 0.2316 |  |  | 0.4361 |
| No | 0.72(0.52,0.98) | 0.0391 | N=10579 | 1.00(0.85,1.17) | 0.9942 | N=10579 | 1.01(0.90,1.13) | 0.8724 | N=10578 | 0.94(0.88,1.01) | 0.1091 | N=10580 |
| Yes | 0.72(0.52,1.00) | 0.0492 | N=8409 | 1.07(0.90,1.27) | 0.4416 | N=8409 | 0.92(0.83,1.02) | 0.1129 | N=8409 | 0.98(0.91,1.05) | 0.5827 | N=8410 |
| STEMI |  |  | 0.9889 |  |  | 0.1802 |  |  | 0.6326 |  |  | 0.8799 |
| No | 0.70(0.43,1.15) | 0.1569 | N=6756 | 0.95(0.80,1.12) | 0.5403 | N=6756 | 0.93(0.84,1.04) | 0.2231 | N=6756 | 0.95(0.88,1.03) | 0.2313 | N=6756 |
| Yes | 0.70(0.54,0.91) | 0.008 | N=12232 | 1.11(0.85,1.30) | 0.1903 | N=12232 | 0.97(0.88,1.07) | 0.5406 | N=12231 | 0.96(0.90,1.03) | 0.2431 | N=12234 |
| Cardiac arrest |  |  | None |  |  | None |  |  | None |  |  | 0.2144 |
| No | 0.74(0.59,0.93) | 0.0092 | N=18807 | 1.04(0.92,1.18) | 0.5055 | N=18807 | 0.96(0.89,1.03) | 0.2887 | N=18806 | 0.86(0.91,1.01) | 0.1274 | N=18809 |
| Yes | None |  | N=181 | None |  | N=181 | None |  | N=181 | 0.77(0.54,1.10) | 0.1495 | N=181 |
| Cardiac shock |  |  | None |  |  | None |  |  | None |  |  | 0.3307 |
| No | 0.74(0.59,0.93) | 0.0096 | N=18560 | 1.04(0.92,1.17) | 0.5672 | N=18560 | 0.97(0.90,1.15) | 0.4855 | N=18649 | 0.97(0.92,1.02) | 0.2217 | N=18562 |
| Yes | None |  | N=338 | None |  | N= 338 | None |  | N=338 | 0.82(0.58,1.15) | 0.2499 | N=338 |
| Heart failure |  |  | 0.0504 |  |  | 0.3537 |  |  | 0.1037 |  |  | 0.0489 |
| No | 0.60(0.47,0.78) | <0.0001 N=17412 | | 1.04(0.91,1.18) | 0.5768 | N=17412 | 0.93(0.86,1.01) | 0.0829 | N=17411 | 0.94(0.89,0.99) | 0.0294 | N=17413 |
| Yes | 1.04(0.64,1.69) | 0.8596 | N=1576 | 0.87(0.62,1.23) | 0.4366 | N=1576 | 1.10(0.91,1.32) | 0.3142 | N=1576 | 1.08(0.95,1.23) | 0.2262 | N=1577 |
| Killip class |  |  | <0.0001 |  |  | 0.3865 |  |  | 0.3893 |  |  | 0.4736 |
| 1 | 0.67(0.52,0.88) | 0.0033 | N=16703 | 1.12(0.97,1.29) | 0.1372 | N=16703 | 0.95(0.87,1.04) | 0.294 | N=16702 | 0.96(0.90,1.02) | 0.1743 | N=16704 |
| 2 | None |  | N=1037 | 0.89(0.48,1.64) | 0.7807 | N=1037 | 0.85(0.68,1.06) | 0.1389 | N=1037 | 0.93(0.79,1.11) | 0.4258 | N=1037 |
| 3 | None |  | N=977 | 0.80(0.55,1.16) | 0.2384 | N=977 | 1.11(0.89,1.37) | 0.352 | N=977 | 1.04(0.90,1.21) | 0.6089 | N=978 |
| 4 | None |  | N=271 | None |  | N=271 | None |  | N=271 | 0.72(0.42,1.21) | 0.2111 | N=271 |
| LVEF category |  |  | 0.8205 |  |  | 0.0247 |  |  | 0..2272 |  |  | 0.3407 |
| 1 | 1.07(0.49,2.33) | 0.8737 | N=2531 | 1.10(0.84,1.43) | 0.4937 | N=2531 | 1.09(0.93,1.27) | 0.3013 | N=2531 | 1.04(0.94,1.16) | 0.4409 | N=2532 |
| 2 | 0.70(0.51,0.94) | 0.194 | N=13370 | 0.95(0.82,1.10) | 0.4812 | N=13370 | 0.90(0.82,0.99) | 0.0288 | N=13370 | 0.93(0.87,0.99) | 0.0322 | N=13371 |
| 3 | None |  | N=1066 | 1.75(0.72,4.25) | 0.2197 | N=1066 | None |  | N=1066 | 1.02(0.78,1.32) | 0.9043 | N=1066 |
| 4 | None |  | N=2021 | 2.42(1.16,5.04) | 0.0183 | N=2021 | 1.01(0.80,1.27) | 0.9249 | N=2020 | 0.95(0.81,1.12) | 0.5653 | N=2021 |
| Angiography |  |  | 0.1729 |  |  | 0.6368 |  |  | 0.6702 |  |  | 0.0661 |
| No | 0.94(0.65,1.37) | 0.7517 | N=7001 | 1.07(0.91,1.25) | 0.3985 | N=7001 | 0.97(0.88,1.06) | 0.4522 | N=7001 | 1.01(0.94,1.07) | 0.8792 | N=7003 |
| Yes | 0.68(0.52,0.89) | 0.0052 | N=11987 | 1.01(0.85,1.20) | 0.8897 | N=11987 | 0.93(0.82,1.06) | 0.5925 | N=11986 | 0.91(0.85,0.99) | 0.0218 | N=11987 |
| PCI |  |  | 0.0987 |  |  | 0.0174 |  |  | 0.1972 |  |  | 0.076 |
| No | 0.89(0.65,1.21) | 0.4523 | N=8912 | 0.94(0.82,1.08) | 0.3621 | N=8912 | 0.98(0.90,1.07) | 0.6649 | N=8912 | 0.99(0.93,1.05) | 0.7587 | N=8914 |
| Yes | 0.61(0.45,0.84) | 0.002 | N=10076 | 1.28(1.03,1.60) | 0.0277 | N=10076 | 0.87(0.74,1.02) | 0.0931 | N=10075 | 0.90(0.82,0.98) | 0.0215 | N=10076 |

Continuity variables, we have three groups of equal divisions from small to large

We used the logistic regression model and interaction test.

**Supplemental Table 3.** The pooled results of multiple imputation.

| Outcomes | beta 1 | se 1 | beta 2 | se 2 | beta 3 | se 3 | beta 4 | se 4 | beta 5 | se 5 |
| --- | --- | --- | --- | --- | --- | --- | --- | --- | --- | --- |
| Major Bleeding | -0.28768 | 0.081238 | -0.27444 | 0.080166 | -0.27444 | 0.080166 | -0.27444 | 0.080166 | -0.27444 | 0.080166 |
| Stoke | 0.019803 | 0.049691 | 0.029559 | 0.04921 | 0.019803 | 0.049691 | 0.029559 | 0.04921 | 0.029559 | 0.051939 |
| CVD death | -0.06188 | 0.024314 | -0.06188 | 0.021724 | -0.06188 | 0.021724 | -0.06188 | 0.021724 | -0.06188 | 0.021724 |
| MACEs | -0.04082 | 0.018707 | -0.05129 | 0.018707 | -0.05129 | 0.018707 | -0.05129 | 0.018707 | -0.05129 | 0.018707 |

| Ootcomes | Coefficients | Se | T | P value | exp (OR) | 95%CI low | 95%CI upp |
| --- | --- | --- | --- | --- | --- | --- | --- |
| Major bleeding | -0.27709 | 0.080599 | -3.43782 | 0.000586 | 0.757989 | 0.647226 | 0.887709 |
| Stroke | 0.025656 | 0.050243 | 0.51064 | 0.609603 | 1.025988 | 0.929767 | 1.132167 |
| CVD death | -0.06188 | 0.022266 | -2.77892 | 0.005454 | 0.94 | 0.899859 | 0.981931 |
| MACEs | -0.0492 | 0.019284 | -2.55126 | 0.010733 | 0.951992 | 0.916681 | 0.988663 |

**Supplemental Table 4. Relationship between baseline hemoglobin level and adverse outcomes in different models before the multiple imputation.**

| Outcomes | Non-adjusted | Adjusted I | Adjusted II |
| --- | --- | --- | --- |
| Major bleeding | 0.73 (0.64, 0.83), *P* < 0.0001, n=20554 | 0.74 (0.64, 0.85), *P* < 0.0001, n=20554 | 0.77 (0.61, 0.97), *P* = 0.0250, n=18267 |
| Stroke | 0.92 (0.85, 1.00), *P* = 0.0427, n=20556 | 1.00 (0.91, 1.10), *P* = 0.9517, n=20556 | 1.03 (0.91, 1.16), *P* = 0.6311, n=18267 |
| CVD death | 0.79 (0.77, 0.82), *P* < 0.0001, n=20553 | 0.89 (0.86, 0.93), *P* < 0.0001, n=20553 | 0.95 (0.88, 1.02), *P* = 0.1500, n=18266 |
| MACE | 0.82 (0.80, 0.85), *P* < 0.0001, n=20559 | 0.91 (0.88, 0.94), *P* < 0.0001, n=20559 | 0.97 (0.92, 1.02), *P* = 0.1859, n=18269 |

MACE: major adverse cardiovascular events. In the Adjusted I model, we adjusted demographic data, including cohort, intervention, age and male sex. In the Adjusted II model, we adjusted all confounders, including cohort, intervention, age, male sex, ST-segment elevation myocardial infarction (STEMI), heartrate, weight, smoking or tobacco, hypertension, peripheral arterial disease (PAD), prior transient ischemic attack (TIA) or stroke, diabetes, cardiac arrest, Killip class, left-ventricular ejection fraction (LVEF) category, minutes from symptom onset to arrival, angiography, percutaneous coronary intervention (PCI).

Multivariate linear regression model was used.

The OR per 1g/dL increase in Hb levels for outcomes.

**Supplemental Table 5. Results of two-piecewise linear-regression model before the multiple imputation.**

|  | Female | Male | Total |
| --- | --- | --- | --- |
| **Major bleeding** | N=4304 | N=13963 | N=18267 |
| One linear-regression model | 0.92 (0.53, 1.60) *P* = 0.7797 | 0.69 (0.52, 0.93) *P* = 0.0130 | 0.77 (0.61, 0.97) *P* = 0.0250 |
| Inflection point (K) | 10 | 13.2 | 13.4 |
| <K Effect size β (95%CI) | None | 0.57 (0.40, 0.82) *P* = 0.0024 | 0.73 (0.55, 0.96) *P* = 0.0255 |
| >K Effect size β (95%CI) | 0.58(0.25, 1.35) *P* = 0.2100 | 1.07 (0.59, 1.92) *P* = 0.8269 | 0.93 (0.52, 1.69) *P* = 0.8192 |
| Log likelihood ratio test | 0.023 | 0.149 | 0.516 |
| **Stroke** | N=4304 | N=13963 | N=18267 |
| One linear-regression model | 1.13 (0.92, 1.40) *P* = 0.2436 | 0.99 (0.86, 1.15) *P* = 0.9436 | 1.03 (0.91, 1.16) *P* = 0.6311 |
| Inflection point (K) | 13.4 | 16.5 | 16.3 |
| <K Effect size β (95%CI) | 1.04 (0.80, 1.35) *P* = 0.7747 | 1.03 (0.88, 1.21) *P* = 0.7147 | 1.05 (0.92, 1.19) *P* = 0.4663 |
| >K Effect size β (95%CI) | 1.53 (0.87, 2.69) *P* = 0.7369 | 0.45 (0.09, 2.39) *P* = 0.3513 | 0.68 (0.22, 2.15) *P* = 0.5137 |
| Log likelihood ratio test | 0.307 | 0.272 | 0.428 |
| **CVD death** | N=4304 | N=13962 | N=18266 |
| One linear-regression model | 0.88 (0.77, 1.01) *P* =0.0612 | 0.97 (0.88, 1.06) *P* =0.5043 | 0.95 (0.88, 1.02) *P* =0.1500 |
| Inflection point (K) | 14.2 | 14.8 | 14.7 |
| <K Effect size β (95%CI) | 0.86 (0.74, 0.98) *P* =0.0259 | 0.90 (0.81, 1.00) *P* =0.0609 | 0.89 (0.82, 0.97) *P* =0.0056 |
| >K Effect size β (95%CI) | 1.45 (0.68, 3.56) *P* =0.2979 | 1.30 (1.01, 1.67) *P* =0.0433 | 1.36 (1.08, 1.70) *P* =0.0080 |
| Log likelihood ratio test | 0.262 | 0.028 | 0.004 |
| **Mace** | N=4305 | N=13964 | N=18269 |
| One linear-regression model | 0.97(0.88, 1.07) *P* = 0.6040 | 0.96 (0.90, 1.02) *P* = 0.2126 | 0.97 (0.92, 1.02) *P* = 0.1859 |
| Inflection point (K) | 13.5 | 13.8 | 13.6 |
| <K Effect size β (95%CI) | 0.93 (0.84, 1.04) *P* = 0.2289 | 0.89 (0.82, 0.97) *P* = 0.0077 | 0.91 (0.85, 0.97) *P* = 0.0043 |
| >K Effect size β (95%CI) | 1.28 (0.90, 1.82) *P* = 0.1761 | 1.10 (0.98, 1.25) *P* = 0.1187 | 1.11 (0.99, 1.24) *P* = 0.0635 |
| Log likelihood ratio test | 0.155 | 0.019 | 0.008 |

MACE: major adverse cardiovascular events, CVD death: cardiovascular death. We adjusted for cohort, intervention, age, male sex, ST-segment elevation myocardial infarction (STEMI), heartrate, weight, smoking or tobacco, hypertension, peripheral arterial disease (PAD), prior transient ischemic attack (TIA) or stroke, diabetes, cardiac arrest, cardiac shock, heart failure, Killip class, left-ventricular ejection fraction (LVEF) category, minutes from symptom onset to arrival , angiography, percutaneous coronary intervention (PCI).

The 2-stage linear regression model was used to calculate the threshold effect.

The OR per 1g/dL increase in Hb levels for outcomes.

**Supplemental Table 6. The R package which is used in the statistical analysis.**

| Statistical method | Logistic regression | GAM | Two-piecewise linear regression | Multiple imputation |
| --- | --- | --- | --- | --- |
| R package | base | mgcv | MASS, segmented | mi |
